# CCDC25 may be a potential diagnostic and prognostic marker of hepatocellular carcinoma: Results from microarray analysis

**DOI:** 10.3389/fsurg.2022.878648

**Published:** 2022-09-23

**Authors:** Hongyang Deng, Jiaxing Zhang, Yijun Zheng, Jipin Li, Qi Xiao, Fengxian Wei, Wei Han, Xiaodong Xu, Youcheng Zhang

**Affiliations:** ^1^ Department of General Surgery, Hepatic-biliary-pancreatic Institute, Lanzhou University Second Hospital, Lanzhou, China; ^2^ Key Laboratory of the Digestive System Tumors of Gansu Province, Lanzhou University Second Hospital, Lanzhou, China

**Keywords:** coiled-coil domain containing 25, hepatocellular carcinoma, prognosis, diagnosis, immune infiltrates, ferroptosis

## Abstract

**Background:**

Hepatocellular carcinoma (HCC) is a tumor with a high recurrence rate, poor prognosis, and rapid progression. Therefore, it is necessary to find a novel biomarker for HCC. Coiled-coil domain containing 25 (CCDC25) has been identified as a target molecule that mediates liver metastasis in colon cancer. However, the molecular mechanisms of CCDC25 in HCC are unknown. This study aimed to explore the role of CCDC25 in HCC.

**Methods:**

The expression of CCDC25 in HCC was identified through The Cancer Genome Atlas (TCGA) and Gene Expression Omnibus (GEO) databases. Receiver operating characteristic curve (ROC) curves were drawn to evaluate the diagnostic value of CCDC25 for HCC. The effect of CCDC25 on the prognosis of HCC was analyzed by using the Kaplan–Meier plotter. Co-expressed genes and Gene Set Enrichment Analysis (GSEA) were used to explore the related functions and regulatory signaling pathways of CCDC25. Moreover, we employed the Tumor Immune Estimation Resource (TIMER) database and CIBERSORT algorithm to investigate the relationship between CCDC25 and the tumor immune microenvironment (TME) in HCC. Meanwhile, the effect of CCDC25 on the sensitivity of HCC patients to chemotherapy drugs was evaluated. Finally, we explored the prognostic methylation sites of CCDC25 using the MethSurv database.

**Results:**

CCDC25 expression was low in HCC. Low CCDC25 expression was significantly associated with poor overall survival of HCC and may be comparable to the ability of AFP to diagnose HCC. Dysregulation of glucose metabolism, fatty acid metabolism, amino acid metabolism, ubiquitination modification, and apoptosis inhibition caused by CCDC25 downregulation may be the causes and results of HCC. In addition, CCDC25 was positively correlated with the infiltration level of various adaptive antitumor immune cells. The levels of immune cell infiltration and immune checkpoint expression were lower in the samples with high CCDC25 expression. What is more, we found that downregulated CCDC25 may increase the sensitivity or resistance of HCC patients to multiple drugs, including sorafenib. We also identified a methylation site for CCDC25, which may be responsible for poor prognosis and low CCDC25 expression in HCC patients. Finally, CCDC25 may be associated with HCC ferroptosis.

**Conclusions:**

CCDC25 may be a potential diagnostic and prognostic marker for HCC and is associated with immune infiltration and ferroptosis.

## Introduction

As the sixth most common cancer, hepatocellular carcinoma (HCC) ranks fourth among cancer deaths (1). According to statistics, 90,600 new HCC patients were diagnosed in 2020, resulting in at least 83,000 deaths (2). At present, the incidence of HCC is still on the rise worldwide (2). On the surface, the 5-year survival rate after local resection or liver transplantation for early HCC patients is approximately 70% (3). However, studies have confirmed that the recurrence rate of HCC after hepatectomy is as high as 50%, and the prognosis of advanced HCC patients is poor (4). What is more, the complex molecular mechanisms of HCC are not yet understood. Therefore, it is necessary to explore new HCC biomarkers to improve the treatment outcome and long-term prognosis of HCC patients.

Coiled-coil domain containing 25 (CCDC25) is located on chromosome 8p, and a group of genes, including CCDC25, is deleted on chromosome 8P in patients with poor HCC prognosis (5). It has been found that CCDC25 overexpression in cholangiocarcinoma (CCA) can enhance the migration ability of CCA cells (6). Later, another study (7) found that the level of CCDC25 in the blood of CCA patients was significantly increased. In addition, CCDC25 is a target molecule (8–10) for the neutrophil extracellular traps (NETs) associated with cancer metastasis. However, the role of CCDC25 in HCC is still lacking.

In this study, the expression of CCDC25 in HCC and its related functional mechanism were explored using multiple online databases and R language, hoping to provide a theoretical basis for the diagnosis and treatment of HCC.

## Materials and methods

### Expression analysis of CCDC25 in pan cancer

We used the Tumor Immune Estimation Resource (TIMER) database (11) (https://cistrome.shinyapps.io/timer/) and the HCC (HCCDB) database (12) (http://lifeome.net/database/hccdb/home.html) to detect the CCDC25 expression in pan cancer. The gene expression profile in the TIMER database is based on The Cancer Genome Atlas (TCGA) database, and the expression levels are calculated in log_2_TPM format. HCCDB (12) is a database integrating 15 HCC public data sets, including Gene Expression Omnibus (GEO), the Liver Hepatocellular Carcinoma Project of TCGA (TCGA-LIHC), and the Liver Cancer-RIKEN JP Project from the International Cancer Genome Consortium (ICGC LIRI-JP).

### Data acquisition and preprocessing

The TCGA-LIHC RNA sequencing data (RNA-seq), consisting of 371 HCC samples and 53 adjacent samples, were downloaded in TPM format from the TCGA database (https://portal.gdc.cancer.gov/), and the batch effects were removed using limma R packages. In addition, we used GSE14520, GSE22058, GSE25097, GSE36376, GSE54236, GSE63898, and GSE64041, which were downloaded from the GEO database (https://www.ncbi.nlm.nih.gov/geo/), to verify the CCDC25 expression level in HCC. The probe ID of each gene was converted to the gene symbol, and the average expression of the same gene symbol was taken. The count format expression profile was converted to TPM format, and log_2_(TPM+1) standardization was performed.

### Clinical correlation analysis

Clinical characteristics and survival information corresponding to the TCGA-LIHC data set were downloaded from the Xena (https://xenabrowser.net/datapages/) database. A total of 365 HCC patients had RNA expression profiles and clinical information. The clinical characteristics included age, sex, race, TNM stage, clinical stage, pathological grade, survival status, and duration of survival.

### Kaplan–Meier plotter and the diagnostic ROC

The Kaplan–Meier plotter (13, 14) (https://kmplot.com/analysis/) database, the data of which emanate from GEO, EGA (European Genome-Phenome Archive), and TCGA, was applied to analyze the relationship between CCDC25 expression and the prognosis of HCC. Log-rank *p*-values <0.05 represented statistical significance. The diagnostic value of CCDC25 was evaluated through diagnostic receiver operating characteristic curve (ROC) of TCGA-LIHC data and six GEO data sets (GSE101685, GSE121248, GSE36376, GSE59259, GSE62232, and GSE76427). We calculated AUC values and plotted ROC curves using the pROC package in R-4.1.2. The diagnostic values of alpha fetoprotein (AFP) and CCDC25 for HCC were compared by using the Delong test.

### Analysis of CCDC25 co-expressed genes and their functional enrichment in HCC

LinkedOmics (15) (http://www.linkedomics.org/login.php) is a public database that contains multiomics data from all 32 TCGA cancer types and 10 Clinical Proteomics Tumor Analysis Consortium (CPTAC) cancer cohorts. The Pearson correlation coefficient was calculated through this database to analyze CCDC25 co-expressed genes, and the results were visualized by using a volcano map and heat map. Genes with a co-expression coefficient greater than 0.2 were analyzed for Gene Ontology (GO) and Kyoto Encyclopedia of Genes and Genomes (KEGG) enrichment.

### Gene set enrichment analysis

Gene Set Enrichment Analysis (16) (GSEA) is a computational method that can be used to determine the effect of co-variation of a set of predefined genes on phenotypic changes. The level of CCDC25 expression was recognized as a phenotypic label. The gene set was permuted 1,000 times for each analysis. KEGG pathways with *p *< 0.05 and false discovery rate (FDR) < 0.25 were considered to be significantly enriched.

### Immune cell infiltration and immune checkpoints

CIBERSORT (17) is an analysis tool for estimating the infiltration degree of 22 kinds of human immune cells by using gene transcriptome data. The TIMER database and CIBERSORT algorithm were used to predict the correlation between CCDC25 expression and tumor-infiltrating immune cells. TISIDB (17) (http://cis.hku.hk/TISIDB/index.php), integrating data from various databases such as Pubmed, TCGA, and DrugBank, is a web portal that analyzes the relationship between tumor and immune system. So, the relationships between CCDC25 expression and the immune checkpoints in HCC were analyzed through TISIDB.

### Drug sensitivity and the methylation site analysis

To investigate the relationship between the sensitivity of HCC patients to chemotherapy drugs and CCDC25 expression, a semi-inhibitory concentration (IC50) of commonly used drugs in the LIHC-STAD cohort was predicted using the “pRRophetic” package. MethSurv (18) (https://biit.cs.ut.ee/methsurv/) is an online database containing information on 25 different cancer types and 7,358 patients in TCGA that can be used for multivariate survival analyses using DNA methylation data. Therefore, MethSurv was used to explore CCDC25 DNA methylation sites associated with survival.

### Statistical method

Data from TCGA and GEO were collated by R-4.1.2. OS, PFS, and DSS were calculated by using the log-rank test. Correlation analysis was performed by using Pearson's correlation test. The Wilcox test was used to compare the differences of continuous variables between the two groups. A value of *p *< 0.05 was considered statistically significant.

## Result

### The expression of CCDC25 in pan cancer and HCC

**Figure 1:**
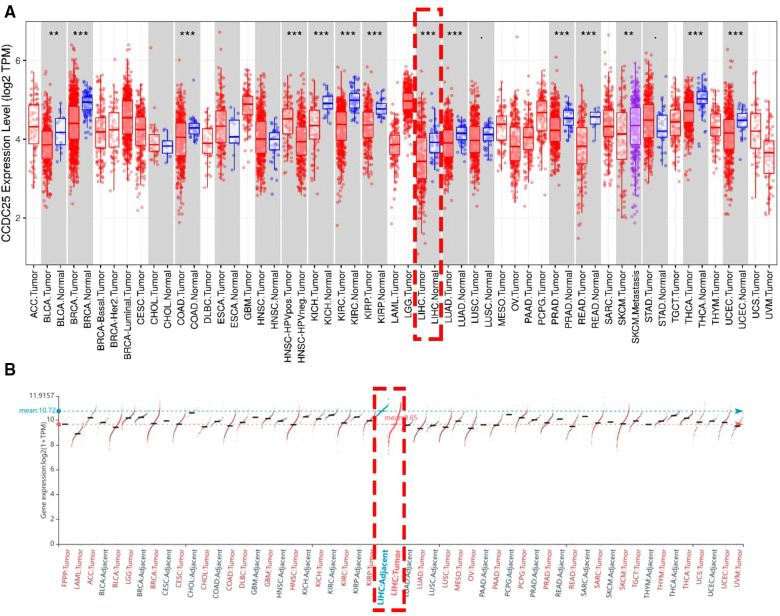
The expression of CCDC25 in pan cancer. (**A**) TIMER, (**B**) HCCDB. Red: HCC tissues, Blue: adjacent tissues. (*p < 0.05; **p < 0.01; ***p < 0.001; ns, not significant, *p* > 0.05.)

**Figure 2:**
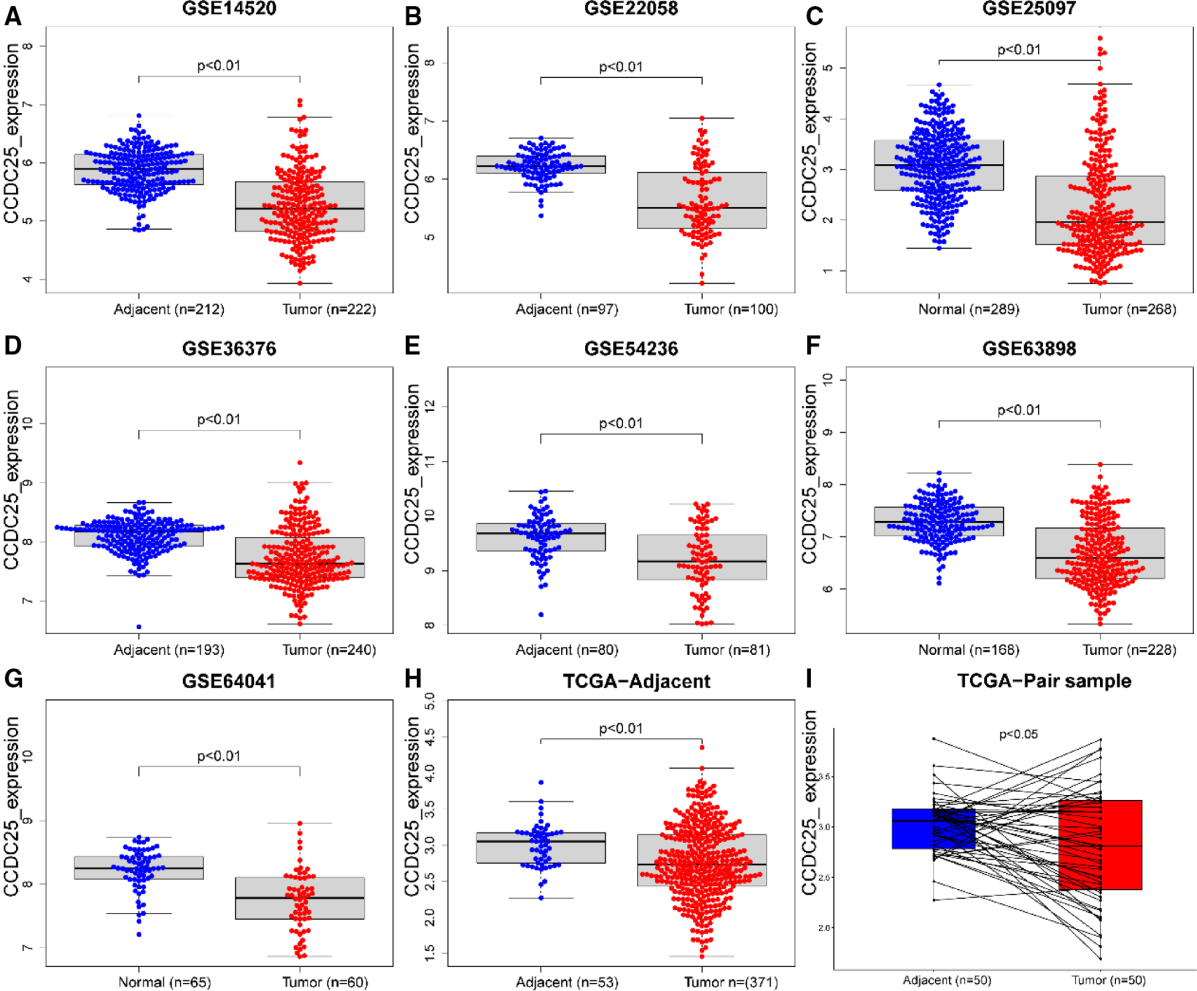
CCDC25 expression in different HCC data sets. (**A**) GSE14520, (**B**)GSE22058, (**C**) GSE25097, (**D**) GSE36376, (**E**) GSE54236, (**F**) GSE63898, (**G**) GSE64041, (**H**) ALL TCGA-LIHC samples, and (**I**) TCGA-LIHC paired samples.

An analysis of CCDC25 expression in pan cancer based on the TIMER database showed that CCDC25 expression levels in bladder urothelial carcinoma (BLCA), breast invasive carcinoma (BRCA), colon adenocarcinoma (COAD), kidney chromophobe (KICH), kidney renal clear cell carcinoma (KIRC), kidney renal papillary cell carcinoma (KIRP), liver hepatocellular carcinoma (LIHC), lung adenocarcinoma (LUAD), prostate adenocarcinoma (PRAD), rectum adenocarcinoma (READ), thyroid carcinoma (THCA), and uterine corpus endometrial carcinoma (UCEC) were significantly lower than those in normal tissues. As for head and neck squamous cell carcinoma (HNSC), CCDC25 expression in samples with HPV^+^ was significantly higher than that in samples with HPV^−^. In addition, the skin cutaneous melanoma (SKCM) with metastasis had a higher CCDC25 expression compared with the SKCM group (Figure 1A). Interestingly, CCDC25 was also significantly underexpressed in HCC in the HCCDB pan-cancer analysis (Figure 1B). This result was confirmed in GSE14520, GSE22058, GSE25097, GSE36376, GSE54236, GSE63898, GSE64041, the TCGA-LIHC data set, and the TCGA-LIHC paired data set (Figure 2). The above results suggested that CCDC25 may be involved in the regulation of a variety of cancers. Particularly in HCC, downregulated CCDC25 may promote the progression of HCC.

### Correlation between CCDC25 expression and clinical characteristics in HCC

**Figure 3:**
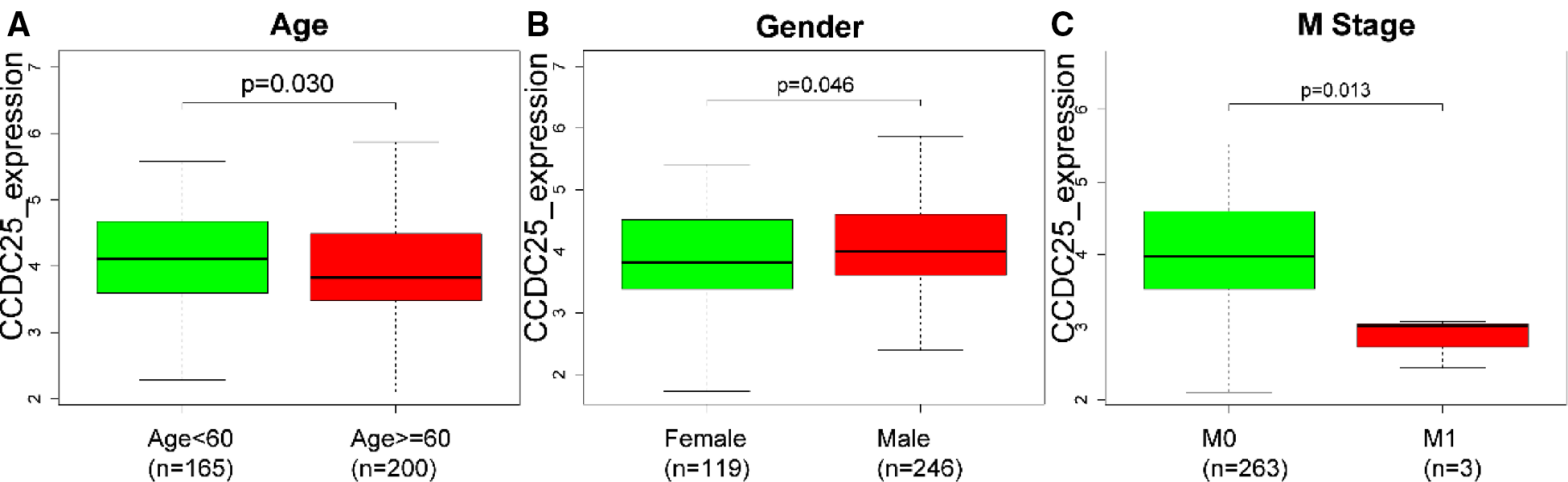
The difference of CCDC25 expression in different clinical subgroups: (**A**) age, (**B**) gender, and (**C**) M stage.

As we can see in Figures 3A–H, downregulated CCDC25 was associated with patients in age (Age < 60 vs. Age ≥ 60, *p *= 0.030), gender (female vs. male, *p *= 0.046), and M stage (M0 vs. M1, *p *= 0.013).

### Diagnostic and prognostic values of CCDC25 in HHC patients

**Figure 4:**
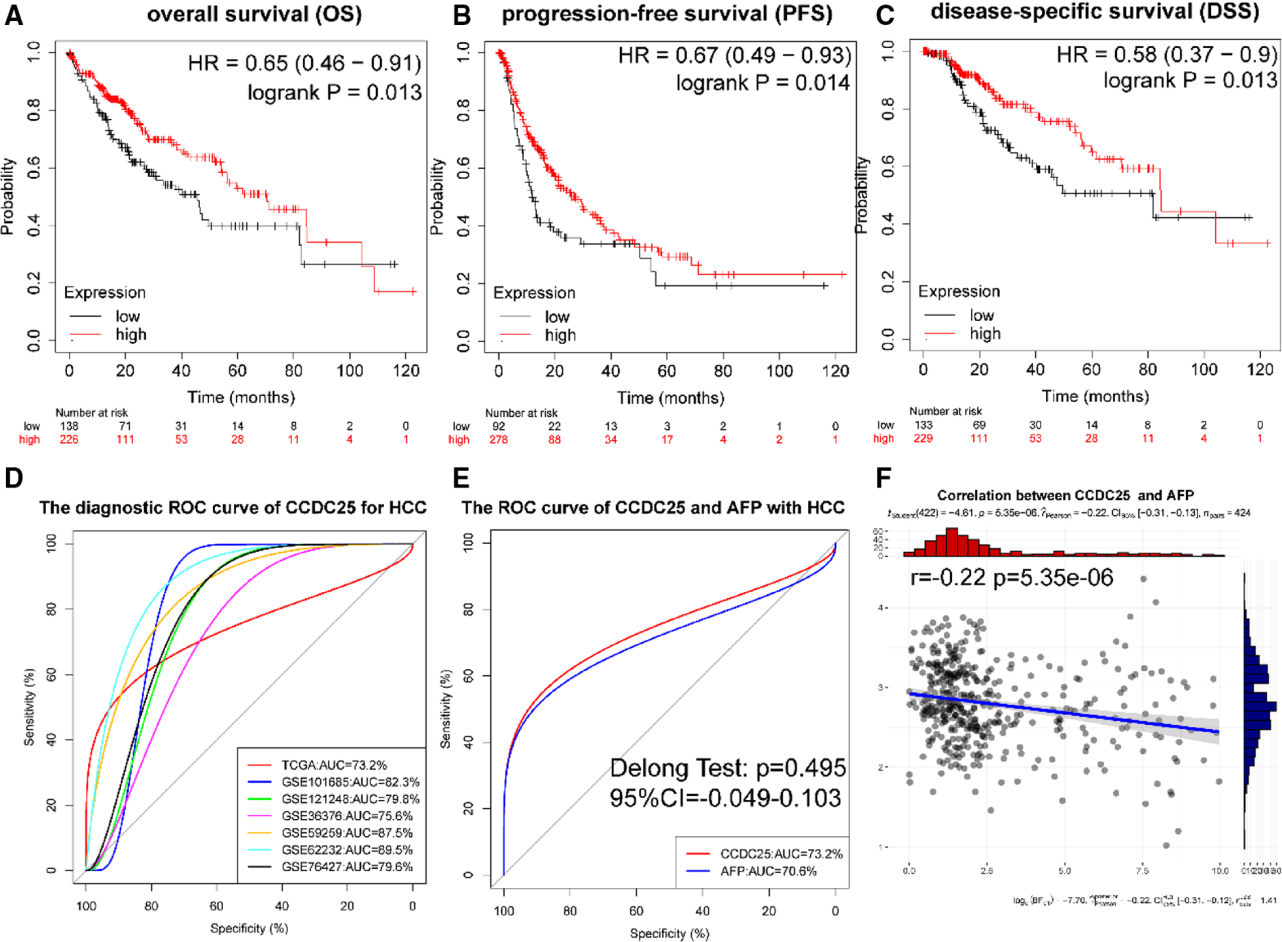
Diagnostic and prognostic values of CCDC25 in HHC patients. (**A**) OS, (**B**) PFS, (**C**) DDS, and (**D**) the ROC curve of CCDC25 diagnostic value for HCC in the TCGA-LIHC and GEO data sets. (**E**) The ROC curve of CCDC25 and AFP diagnostic value of HCC in TCGA-LIHC. (**F**) The correlation between CCDC25 and AFP in TCGA-LIHC.

Figures 4A–C show that a lower CCDC25 expression was significantly related to poor overall survival (OS) (*p *= 0.013), progression-free survival (PFS) (*p *= 0.014), and disease-specific survival (DSS) (*p *= 0.013). As shown in Figure 4D, CCDC25 expression had a strong diagnostic value for HCC (the AUC values of TCGA-LIHC, GSE101685, GSE121248, GSE36376, GSE59259, GSE62232, and GSE76427 were 0.732, 0.823, 0.798, 0.756, 0.875, 0.895, and 0.796). In addition, the diagnostic value of CCDC25 was similar to that of AFP (AUC: 0.723 vs. 0.706, *p *> 0.05) (Figure 4E), and CCDC25 was significantly negatively correlated with AFP (Figure 4F). These results suggested that CCDC25 expression may be a potential predictor of prognosis and diagnosis in HCC.

### Analysis of the CCDC25 co-expressed gene network

**Figure 5:**
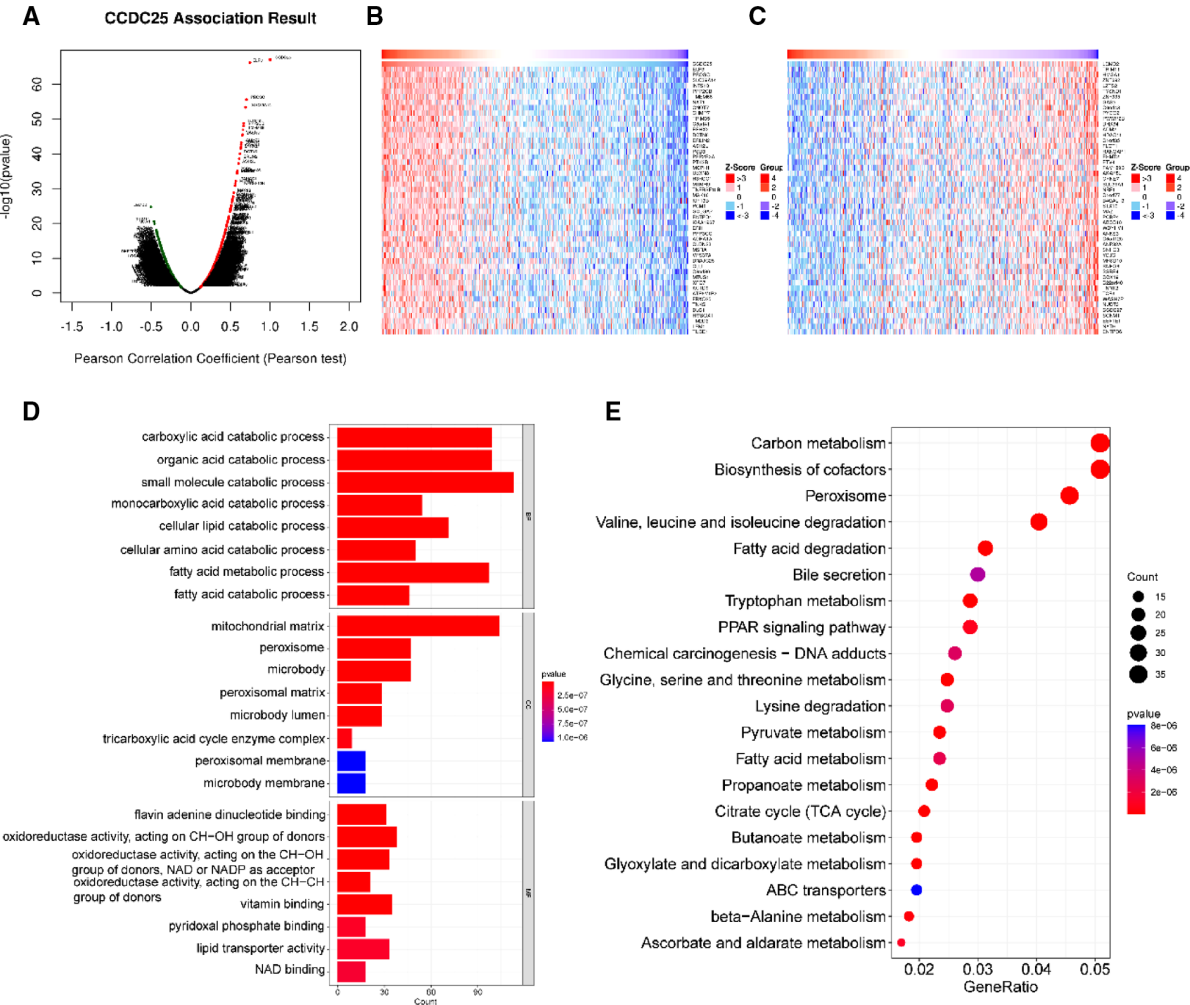
Function enrichment analysis of CCDC25 co-expressed genes. (**A**) The volcanic map shows the co-expression genes of CCDC25 in HCC, (**B**) the top 50 genes positively correlate with CCDC25, (**C**) the top 50 genes negatively correlate with CCDC25, (**D**) GO enrichment analysis for CCDC25 co-expressed genes, and (**E**) KEGG enrichment analysis for CCDC25 co-expressed genes.

With a correlation coefficient greater than 0.2, we identified 1,557 genes that were significantly positively correlated with CCDC25. The heat map shows the top 50 genes with the most significant positive and negative associations with CCDC25 (Figures 5A–C). GO and KEGG enrichment analysis of 1,557 positively related genes showed that CCDC25 expression was mainly involved in metabolism-related functions such as the citrate cycle, fatty acid metabolism, amino acid metabolism, and PPAR signaling pathway (Figures 5D,E). These results suggested that CCDC25 was mainly involved in liver energy metabolism, and its downregulated expression may cause metabolic disorders, thereby promoting the occurrence of HCC.

### Gene set enrichment analysis

**Figure 6:**
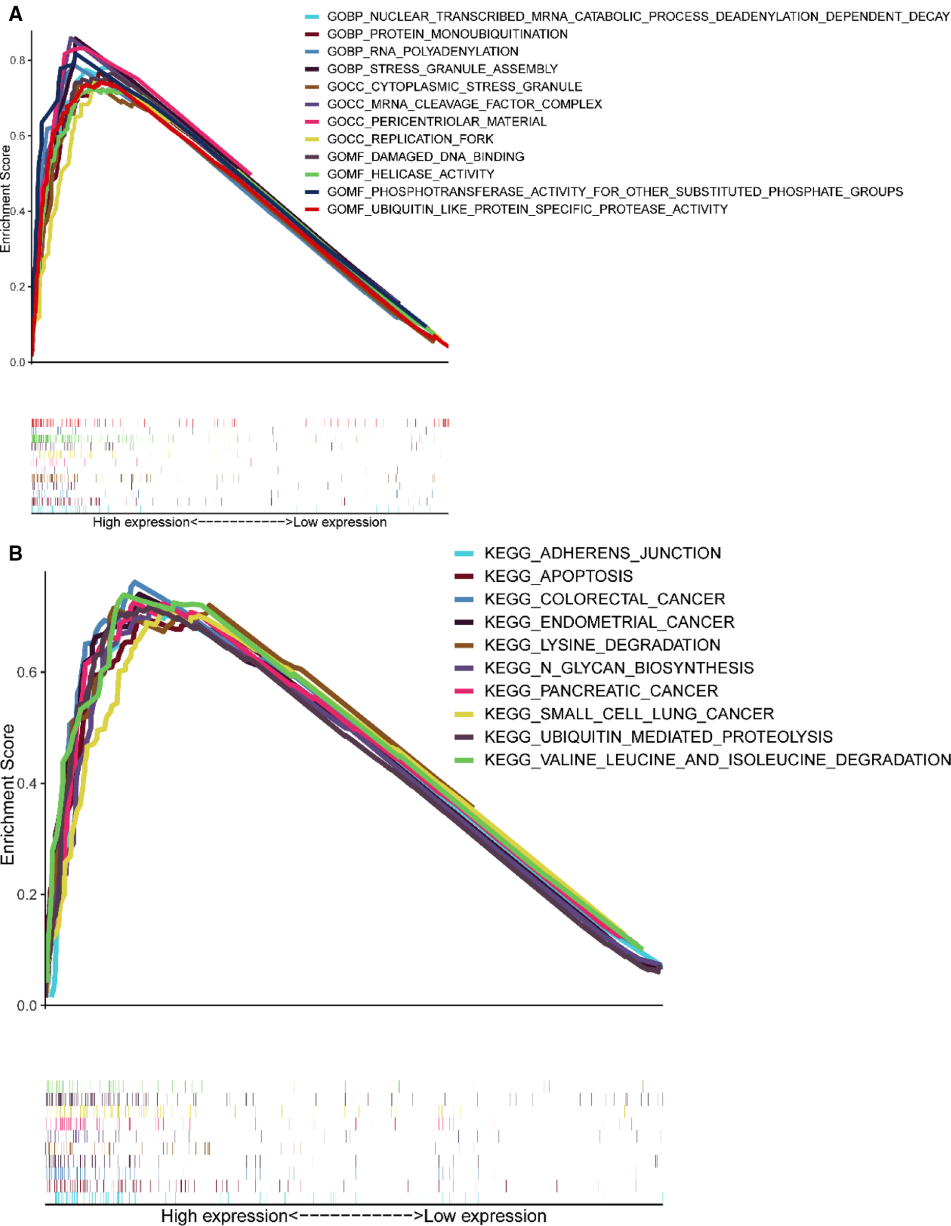
Enrichment plots of GSEA. (**A**) The results of GO enrichment. (**B**) The top 10 results of KEGG enrichment.

GSEA results showed (Figure 6) that CCDC25 was related to the nuclear-transcribed mRNA catabolic process, deadenylation-dependent decay, RNA polyadenylation, helicase activity, ubiquitin-like protein-specific protease activity, damaged DNA binding, adherens junction, apoptosis, lysine degradation, ubiquitin-mediated proteolysis, and valine, leucine, and isoleucine degradation (Figure 6), indicating that CCDC25 is involved in a variety of metabolisms, nucleotide metabolisms, and the promotion of apoptosis processes in the body to maintain normal functions. When CCDC25 expression level is downregulated, abnormalities of these processes may lead to the occurrence of HCC.

### Correlation of CCDC25 expression with immune cell infiltration and immune checkpoints

**Figure 7:**
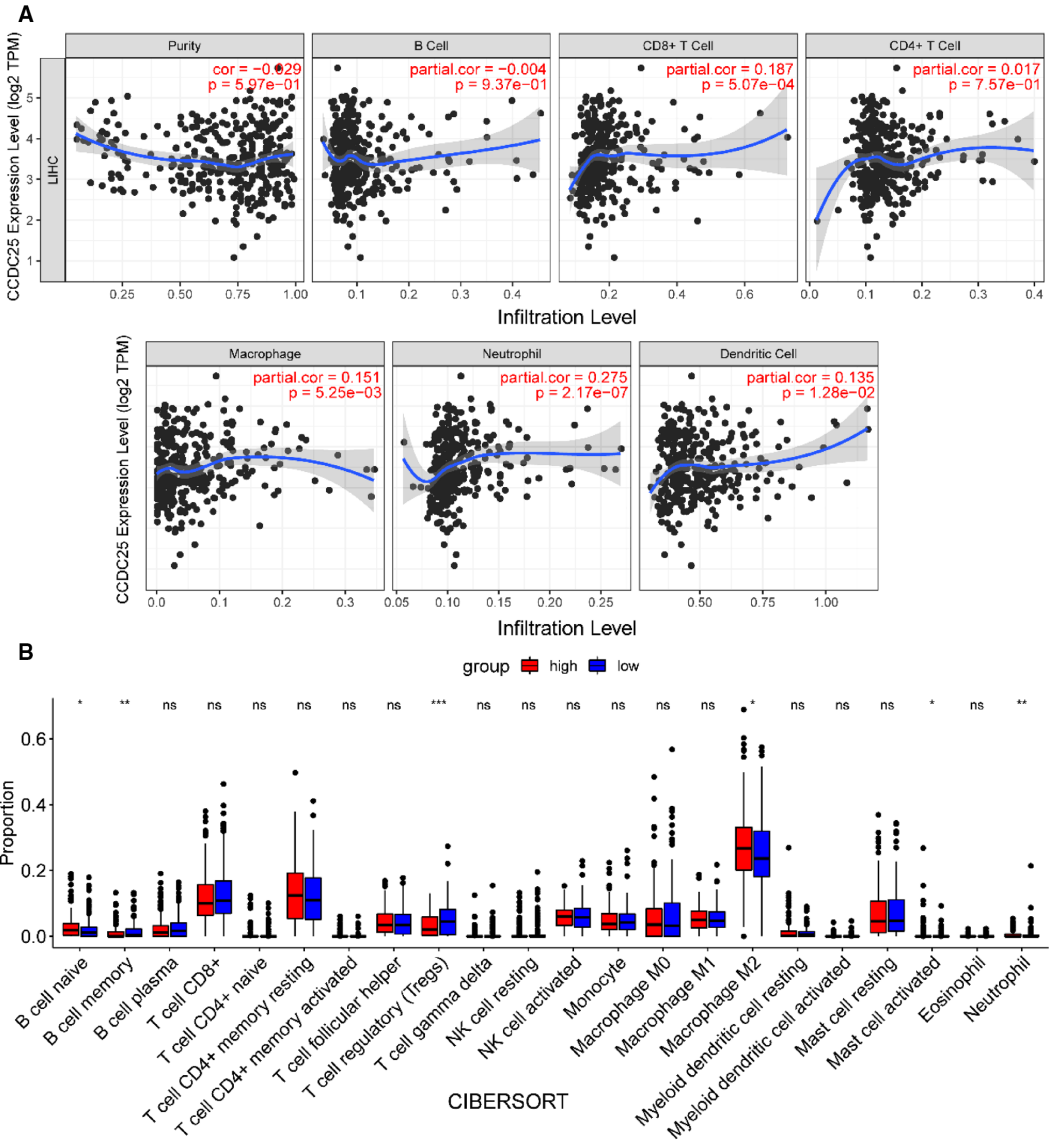
CCDC25 affects immune cell infiltration in HCC patients. (**A**) CCDC25 expression is significantly correlated with the TIMER immune cell infiltration level. (**B**) The boxplot shows the relationship between CCDC25 expression and the abundance of 22 kinds of immune cells in HCC.

**Figure 8:**
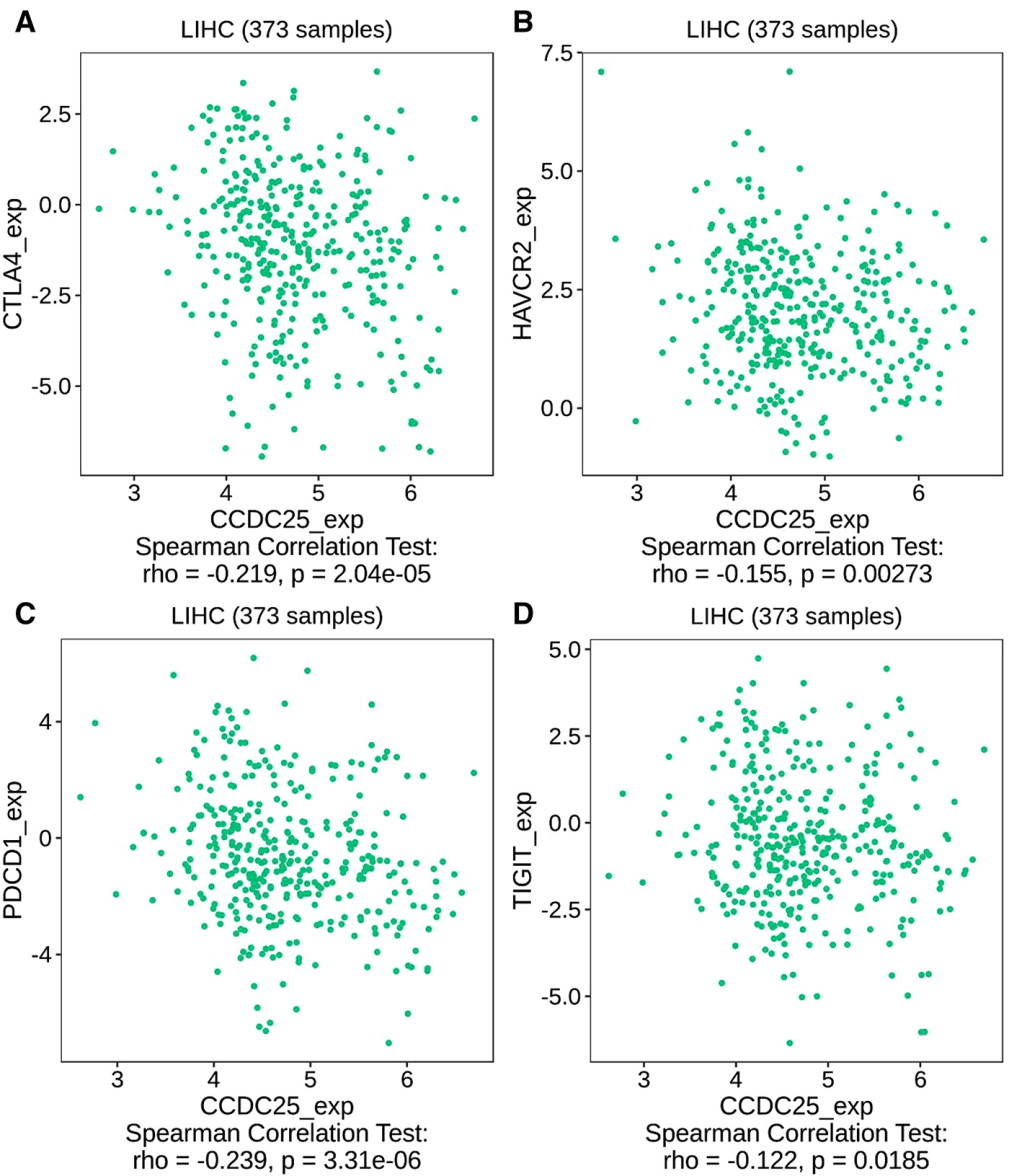
The relationship between CCDC25 expression and the immune checkpoints of HCC. (**A**) CTLA4, (**B**) HAVCR2, (**C**) PDCD1, and (**D**) TIGIT.

We comprehensively evaluated the biological role of CCDC25 in TME through TIMER (Figure 7A) and found that the CD8 + T cell, macrophage, neutrophil, and dendritic cells were positively correlated with CCDC25 expression in HCC. The gene expression profile of TCGA-LIHC was then processed by using the CIBERSORT algorithm to explore the difference in 22 immune cell infiltration between the high CCDC25 expression group and the low CCDC25 expression group in HCC. The results show that the naïve B cell, M2 macrophage, activated mast cell, and neutrophil were elevated in the high CCDC25 expression cohort compared with the low one. Contrastively, the proportion of memory B cells and regulatory T cells (Treg) in the group with high CCDC25 expression was significantly lower than that in the group with low CCDC25 expression (Figure 7B). In addition, CCDC25 expression was found to be negatively correlated with the immune checkpoints PDCD1, CTLA4, TIGIT, and TIM3 (HAVCR2) by TISIDB (Figure 8). These findings suggested that CCDC25 expression may promote antitumor immunity by upregulating CD8 + T cells and dendritic cells and downregulating Treg cells, and it may inhibit tumor immune escape by inhibiting immune checkpoint expression.

### Drug sensitivity analysis

**Figure 9:**
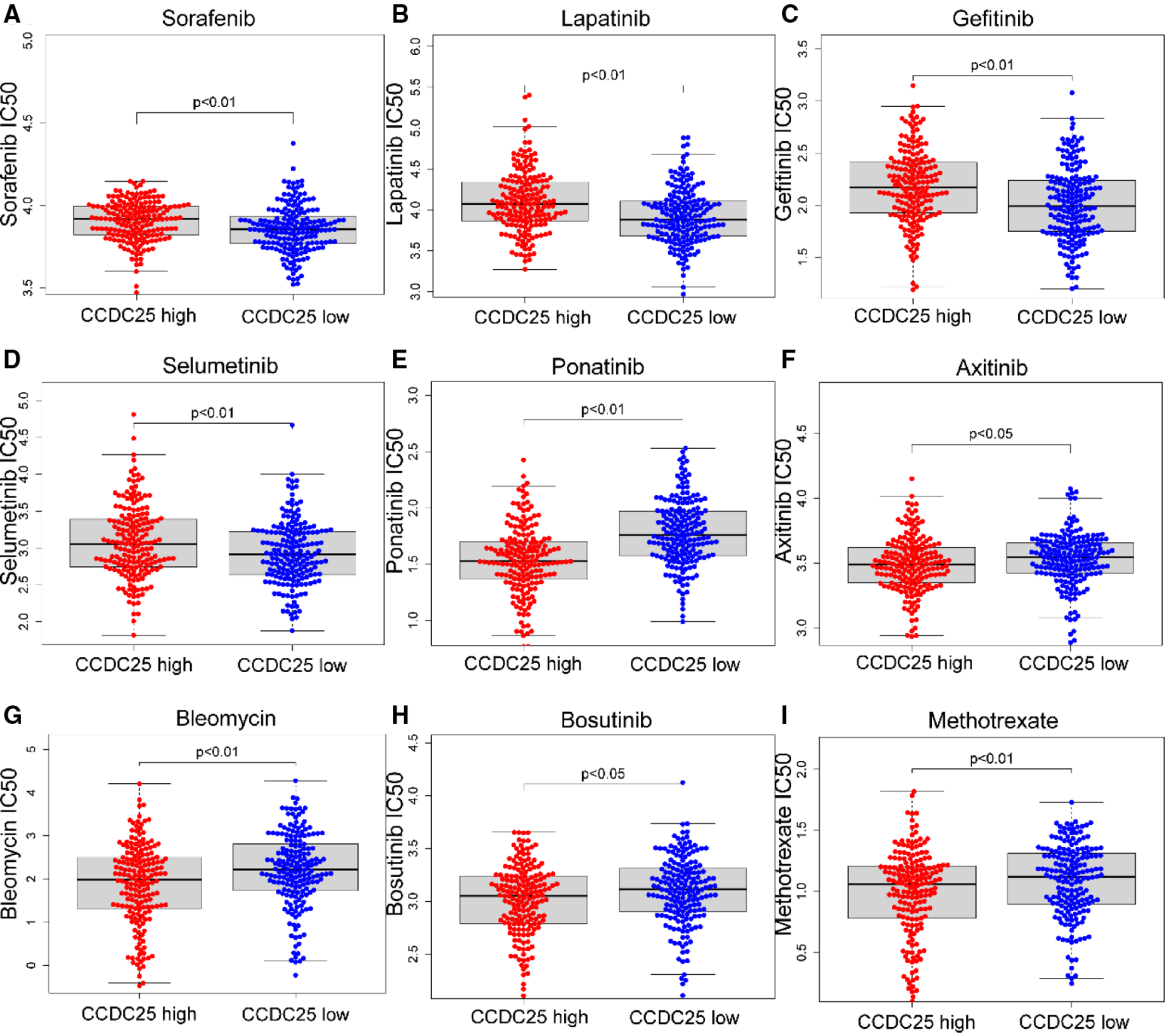
Relationship between chemotherapeutic drug sensitivity and CCDC25 expression in HCC. (**A**) Sorafenib, (**B**) Lapatinib, (**C**) Gefitinib, (**D**) Selumetinib, (**E**) Ponatinib, (**F**) Axitinib, (**G**) Bleomycin, (**H**) Bosutinib, and (**I**) Methotrexate.

For patients with unresectable HCC, chemotherapy and targeted therapy may improve patient outcomes (19). Sorafenib is currently the first-line chemotherapy for HCC patients, but the tolerability of sorafenib shown by many patients remains a major challenge (20). To identify the efficacy of CCDC25 as a biomarker for predicting treatment response in HCC patients, we evaluated the IC50 of 138 drugs in TCGA-LIHC patients. We discovered that patients with low CCDC25 expression may be more sensitive to Sorafenib, Lapatinib, Gefitinib, and Selumetinib, while patients with high CCDC25 expression may respond better to Ponatinib, Axitinib, Bleomycin, Bosutinib, and Methotrexate (Figure 9). Taken together, these results suggested that CCDC25 was associated with a drug sensitivity of HCC, and patients with low CCDC25 expression may have high sensitivity to Sorafenib and may be expected to help in the sensitization treatment of Sorafenib.

### The methylation site of CCDC25

**Figure 10:**
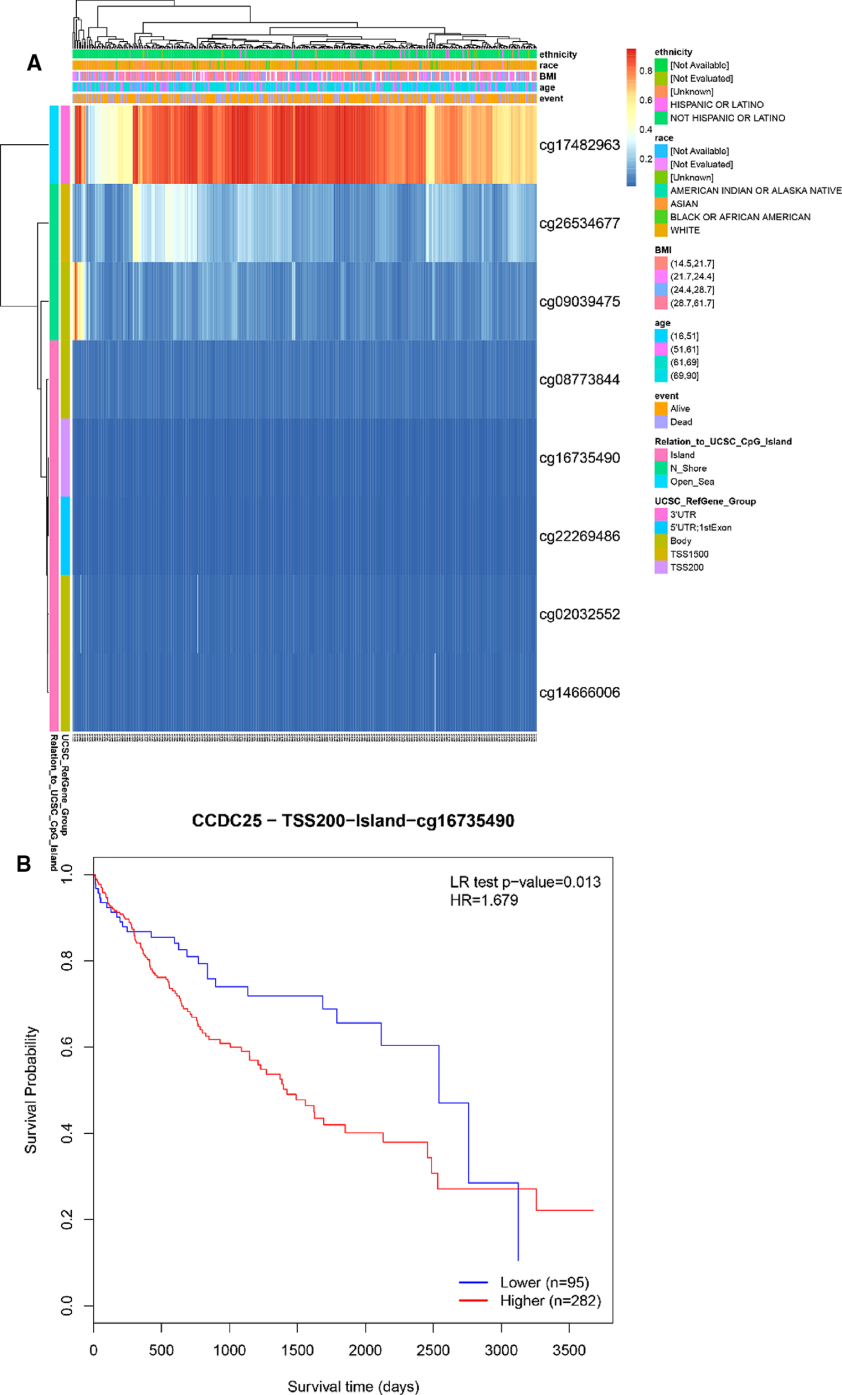
DNA methylation sites of CCDC25. (**A**) Blue to red indicates low to high levels of methylation. The different colored boxes represent ethnicity, race, Body Mass Index (BMI), age, event, relation to UCSC_CpG_Island, UCSC_RefGene_Group. (**B**) The HCC survival analysis of CCDC25-TSS200-Island-cg16735490.

We identified eight DNA methylation sites of CCDC25. Among them, CCDC25-TSS200-Island-cg16735490 was significantly positively related to a shorter survival time (HR = 1.679, *p *= 0.013) (Figure 10). These results supported previous findings that HCC patients with low CCDC25 expression had a poorer prognosis, and the low CCDC25 expression may be associated with hypermethylation at site CCDC25-TSS200-Island-cg16735490.

### CCDC25 expression may be associated with ferroptosis in HCC

**Figure 11:**
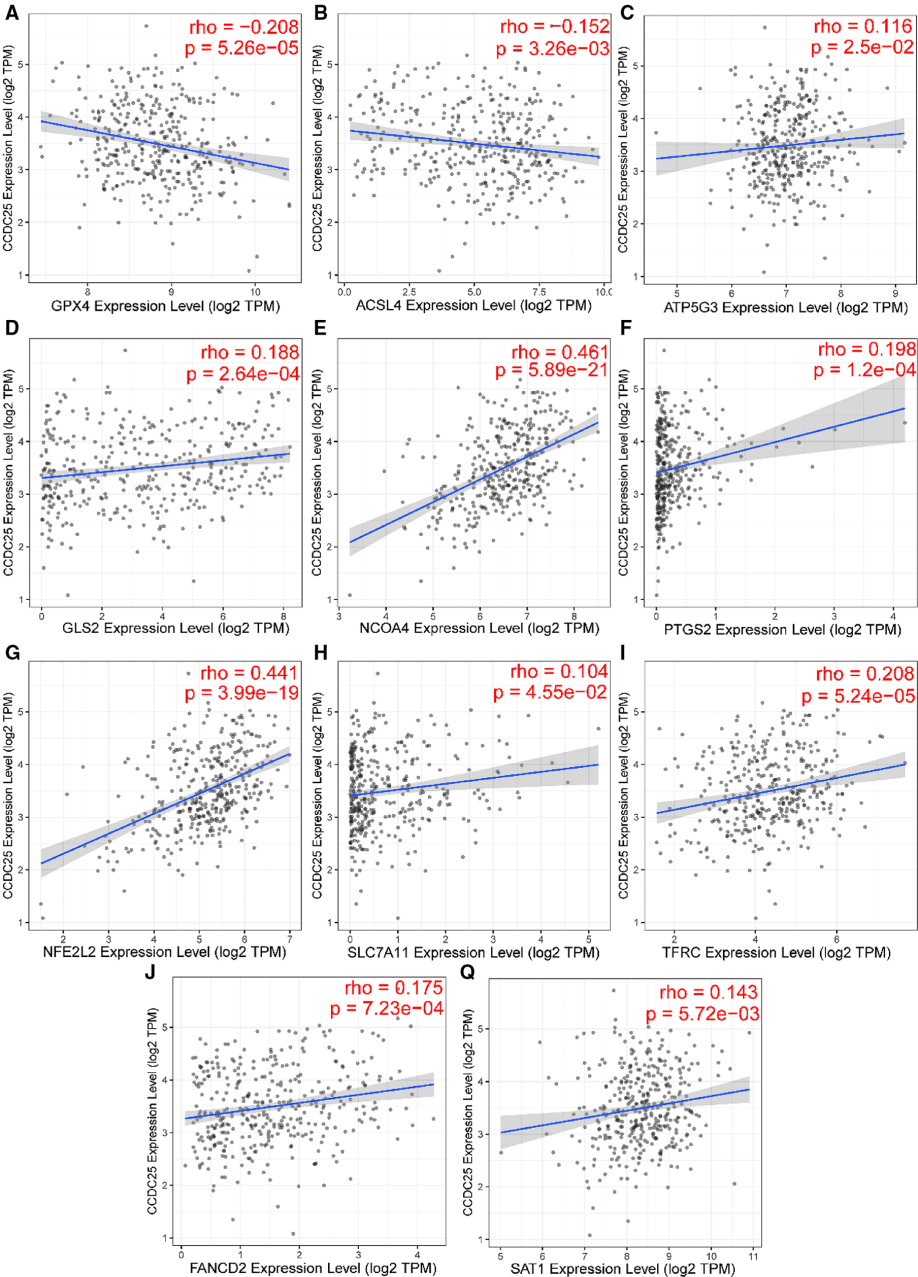
The relationship between CCDC25 expression and ferroptosis key enzymes (**A**) GPX4, (**B**) ACSL4, (**C**) ATP5G3, (**D**) GLS2, (**E**) NCOA4, (**F**) PTGS2, (**G**) NFE2L2, (**H**) SLC7A11, (**I**) TFRC, (**J**) FANCD2, and (**Q**) SAT1.

Apoptosis is a classical form of cell death and plays an important role in maintaining metabolism. In the above analysis, CCDC25 was found to be involved in apoptosis. Ferroptosis, as a new type of regulatory cell death, has been reported to have negative effects on cancer (21). Therefore, to investigate the role of CCDC25 in the ferroptosis of HCC cells, we analyzed the correlation between CCDC25 and the expression levels of various key enzymes of ferroptosis through the TIMER database. The results suggested that CCDC25 was significantly negatively correlated with GPX4 (cor = −0.208, *p *= 5.26 × 10^−5^) and ACSL4 (cor = −0.152, *p *= 3.26 × 10^−3^) and was significantly positively correlated with ATP5G3 (cor = 0.116, *p *= 2.5 × 10^−2^), GLS2 (cor = 0.188, *p *= 2.64 × 10^−4^), NCOA4 (cor = 0.461, *p *= 0.589 × 10^−21^), PTGS2 (cor = 0.198, *p *= 1.2 × 10^−4^), NFE2L2 (cor = 0.441, *p *= 3.99 × 10^−19^), SLC7A11 (cor = 0.104, *p *= 4.55 × 10^−2^), TFRC (cor = 0.208, *p *= 5.24 × 10^−5^), FANCD2 (cor = 0.175, *p *= 7.23 × 10^−4^), and SAT1 (cor = 0.143, *p *= 5.72 × 10^−3^) (Figure 11 and Table 1). These results suggested that CCDC25 may not only participate in apoptosis, but also play an antitumor role by regulating ferroptosis.

**Table 1:** The relationship between CCDC25 and ferroptosis key enzymes

Key enzymes of ferroptosis	Pearson correlation coefficient	*p*-Value
GPX4	−0.208	5.28 × 10^−5^
ACSL4	−0.152	3.26 × 10^−3^
ATP5G3	0.116	2.50 × 10^−2^
GLS2	0.188	2.64 × 10^−4^
NCOA4	0.461	5.89 × 10^−21^
PTGS2	0.198	1.20 × 10^−4^
NFE2L2	0.441	3.99 × 10^−19^
SLC7A11	0.104	4.55 × 10^−2^
TFRC	0.208	5.24 × 10^−5^
FANCD2	0.175	7.23 × 10^−4^
SAT1	0.143	5.72 × 10^−3^

## Discussion

HCC is a serious global health problem, and Asia is particularly vulnerable to HCC as a region with a high incidence of HBV infection (22). The incidence of HCC will continue to increase in the coming decades (23). Therefore, it is necessary to find biomarkers that can be used for early diagnosis or as therapeutic targets for HCC, which may throw new light into the diagnosis and treatment of the disease.

Compared with normal tissues, CCDC25 expression was significantly reduced in HCC tissues of seven GEO data sets, which was confirmed by the TCGA-LIHC data. At the same time, its expression was reduced in many tumors. We suspected that it may play an inhibitory role in HCC or may play a protective role in normal liver tissue. Subsequently, we analyzed the relationship between CCDC25 expression and prognosis of HCC patients and found that HCC patients with low CCDC25 expression had a poor prognosis, which was consistent with our conjecture. Meanwhile, the calculation results of multiple data sets indicated that CCDC25 had a good diagnostic ability for HCC, and its diagnostic value was comparable to that of APF. Based on the above analysis, we have reason to believe that CCDC25 may be a promising prognostic indicator in HCC.

Co-expressed genes are generally involved in similar biological functions, so we explored the co-expressed gene network of CCDC25 in HCC through LinkedOmics. The enrichment analysis suggested that CCDC25 was mainly involved in the citrate cycle, fatty acid metabolism, amino acid metabolism, and PPAR signaling pathway. The citric acid cycle is the body’s main way of obtaining ATP, and cancer cells seem to prefer aerobic glycolysis, which is known as the Warburg effect (24, 25). If CCDC25 is involved in the citrate cycle, to which cancer cells are not prone, it may explain its downregulation in HCC. Many studies have shown that lipid (26–28) and amino acid (29) metabolism disorders are closely related to the occurrence and progression of HCC. Low CCDC25 expression may promote metabolic reprogramming and thus contribute to the development of HCC. The PPAR signaling pathway (30) mainly includes members of the peroxisome proliferator-activated receptor family, which is the main regulator of glucose and lipid metabolism. In conclusion, CCDC25 with a high expression in normal liver tissue may be involved in three metabolic pathways to maintain body function, and its downregulation may promote metabolic reprogramming in favor of HCC.

In addition, the GSEA results showed that CCDC25 was associated with nucleotide metabolism, apoptosis, ubiquitination modification, and DNA damage binding. Controlled cell growth and programmed death are keys to homeostasis. Apoptosis is important for the elimination of excess, virus-infected, and damaged cells (31). CCDC25 may be a tumor suppressor gene that mediates apoptosis. Protein ubiquitination is a form of post-translational modification that regulates protein activity. It involves protein degradation, DNA damage repair, gene transcription, and signal transduction, and the dysregulation of this process is closely related to cancer (32). In conclusion, we found that CCDC25 may mainly play a role in metabolism, epigenetic modification, and regulation of programmed cell death. In conclusion, this study is the first to report that downregulated CCDC25 may inhibit the above-mentioned pathways and promote the occurrence and development of HCC.

The relationship between the TME and the cancer cells is very complex (33), and TME is closely related to tumor cell proliferation, apoptosis, and metastasis (34). It has been argued (35) that there are fundamental differences in the composition of immune cells in healthy tissue, adjacent tissue, and cancer tissue. A number of studies (36) have shown that B cells play an antitumor role by directly interacting with tumor cells or assisting other immune functions. CD8 + T cells are the main antitumor effector cells, while Treg cells often suppress antitumor immunity (37). We found that CCDC25 seems to increase antitumor immunity by increasing CD8 + T cells and decreasing Treg cells. Yang et al. reported (8) that the DNA of NETs, which are released by neutrophils, promotes cancer metastasis. Our findings suggested that patients with a high CCDC25 expression indeed had more neutrophil infiltration. Therefore, CCDC25 may negatively affect HCC by changing the degree of immune cell infiltration in TME.

It has been demonstrated that PDCD-1 (38), TIGIT (39), CTLA-4 (40), and TIM-3 (41) help tumor cell immune evasion through different mechanisms. Our study found that CCDC25 expression was negatively correlated with these four immune checkpoints in HCC, suggesting that CCDC25 may negatively regulate these immune checkpoints to play an antitumor role.

Sorafenib (42) is the first first-line chemotherapy agent for advanced HCC approved by the US Food and Drug Administration (FDA). However, advanced HCC patients will fail treatment due to sorafenib tolerance (22). In this study, we found that HCC patients with high CCDC25 expression may have higher sorafenib tolerance, and patients with low CCDC25 expression may have better sensitivity to sorafenib treatment. In addition, we found that patients with low CCDC25 expression had better sensitivity to Lapatinib, Gefitinib, and Selumetinib, while Ponatinib, Axitinib, Bleomycin, Bosutinib, and Methotrexate may be potentially sensitive drugs for HCC patients with a high expression of CCDC25.

Ferroptosis (43) is a newly discovered type of programmed cell death characterized by iron-dependence lipid peroxidation. Emerging evidence shows that ferroptosis is involved in various liver diseases, especially HCC (44). Glutathione peroxidase 4 (GPX4) (45) has been shown to inhibit ferroptosis in cancer cells. The decreased activity of GPX4 (46) can hinder the metabolism of lipid peroxides, which leads to a breakdown of cell membrane integrity and ultimately ferroptosis. Interestingly, GPX4 played a carcinogenic role in HCC and was negatively correlated with CCDC25 in this study. Subsequently, we found that CCDC25 was associated with many ferroptosis key enzymes, which suggested that CCDC25 might be a key molecule in the development of ferroptosis.

This study is the first to reveal the relationship between CCDC25 and HCC; it suggested that CCDC25 might have a complex impact on the prognosis, diagnosis, and TME in HCC. In conclusion, CCDC25 is expected to be a promising diagnostic and prognostic biomarker for HCC patients, and its function in HCC deserves further exploration.

## Data Sharing Statement

All data generated or analyzed during this study are available in the TCGA database (https://portal.gdc.cancer.gov) and GEO database (https://www.ncbi.nlm.nih.gov/geo/).

## Data Availability

Publicly available data sets were analyzed in this study. These data sets can be found in https://portal.gdc.cancer.gov https://www.ncbi.nlm.nih.gov/geo/.
