# Corrigendum: CCDC25 may be a potential diagnostic and prognostic marker of hepatocellular carcinoma: Results from microarray analysis

**DOI:** 10.3389/fsurg.2023.1175721

**Published:** 2023-03-28

**Authors:** Hongyang Deng, Jiaxing Zhang, Yijun Zheng, Jipin Li, Qi Xiao, Fengxian Wei, Wei Han, Xiaodong Xu, Youcheng Zhang

**Affiliations:** ^1^ Department of General Surgery, Hepatic-Biliary-Pancreatic Institute, Lanzhou University Second Hospital, Lanzhou, China; ^2^ Key Laboratory of the Digestive System Tumors of Gansu Province, Lanzhou University Second Hospital, Lanzhou, China

**Keywords:** coiled-coil domain containing 25, hepatocellular carcinoma, prognosis, diagnosis, immune infiltrates, ferroptosis

A Corrigendum on CCDC25 may be a potential diagnostic and prognostic marker of hepatocellular carcinoma: Results from microarray analysis By Deng H, Zhang J, Zheng Y, Li J, Xiao Q, Wei F, Han W, Xu X, Zhang Y. (2023) Front. Surg. 10:878648. doi: 10.3389/fsurg.2022.878648

**Text Correction**

In the published article, there was an error. A text error occurred in the email address of correspondence.

A correction has been made to the email address of correspondence. This sentence previously stated:

**zhangychmd@126.csom**

The corrected sentence appears below: **zhangychmd@126.com**

The authors apologize for this error and state that this does not change the scientific conclusions of the article in any way. The original article has been updated.

